# Machine learning-based preoperative datamining can predict the therapeutic outcome of sleep surgery in OSA subjects

**DOI:** 10.1038/s41598-021-94454-4

**Published:** 2021-07-21

**Authors:** Jin Youp Kim, Hyoun-Joong Kong, Su Hwan Kim, Sangjun Lee, Seung Heon Kang, Seung Cheol Han, Do Won Kim, Jeong-Yeon Ji, Hyun Jik Kim

**Affiliations:** 1 Department of Otorhinolaryngology–Head and Neck Surgery, Ilsan Hospital, Dongguk University, Goyang, Gyeonggi Korea; 2 Interdisciplinary Program of Medical Informatics, Seoul National University College of Medicine, Seoul, Korea; 3 Transdisciplinary Department of Medicine & Advanced Technology, Seoul National University Hospital, Seoul, Korea; 4 Medical Research Center, Institute of Medical and Biological Engineering, Seoul National University, Seoul, Korea; 5 Department of Biomedical Engineering, Seoul National University College of Medicine, Seoul, Korea; 6 Department of Preventive Medicine, Seoul National University College of Medicine, Seoul, Korea; 7 Department of Biomedical Sciences, Seoul National University College of Medicine, Seoul, Korea; 8 Department of Otorhinolaryngology - Head and Neck Surgery, Seoul National University College of Medicine, 103, Daehak-ro, Jongno-gu, Seoul, 03080 Republic of Korea

**Keywords:** Computational biology and bioinformatics, Medical research

## Abstract

Increasing recognition of anatomical obstruction has resulted in a large variety of sleep surgeries to improve anatomic collapse of obstructive sleep apnea (OSA) and the prediction of whether sleep surgery will have successful outcome is very important. The aim of this study is to assess a machine learning-based clinical model that predict the success rate of sleep surgery in OSA subjects. The predicted success rate from machine learning and the predicted subjective surgical outcome from the physician were compared with the actual success rate in 163 male dominated-OSA subjects. Predicted success rate of sleep surgery from machine learning models based on sleep parameters and endoscopic findings of upper airway demonstrated higher accuracy than subjective predicted value of sleep surgeon. The gradient boosting model showed the best performance to predict the surgical success that is evaluated by pre- and post-operative polysomnography or home sleep apnea testing among the logistic regression and three machine learning models, and the accuracy of gradient boosting model (0.708) was significantly higher than logistic regression model (0.542). Our data demonstrate that the data mining-driven prediction such as gradient boosting exhibited higher accuracy for prediction of surgical outcome and we can provide accurate information on surgical outcomes before surgery to OSA subjects using machine learning models.

## Introduction

The excessive upper airway narrowing reportedly contributes to the underlying pathogenesis of obstructive sleep apnea (OSA) and leads to sleep-related symptoms or systemic cardiovascular complications if not properly treated^1–8^. The upper airway narrowing can be caused by collapse at multiple levels, such as the soft palate, uvula, palatine tonsils, lateral pharyngeal walls, and tongue base^3,9^. So far, diverse sleep surgeries have been developed to correct the upper airway narrowing of OSA subjects and the focus on enhancing the tension of the upper airway muscles resulting in widening the pharyngeal lumen^10–15^.

The success rate of sleep surgery is known to vary widely, ranging from 45 to 78% in OSA subjects^10–12^ and it is essential to determine OSA subjects suitable for sleep surgery to avoid unnecessary surgical treatment and to provide optimal surgery to OSA subjects. In particular, sleep surgery could be more effective if it is possible to know in advance how successful the surgery will be prior to surgery. Until now, prediction of the success rate of sleep surgery has depended on the subjective experience of the sleep surgeons, and objective prediction based on the results of polysomnography (PSG) or the subjects’ upper airway findings has not been attempted much in the clinical field.

The development of a screening model based on clinical features collected from OSA subjects and sleep parameters would be extremely practical in predicting therapeutic outcomes. Such a model can also help sleep physicians provide more adequate therapeutic options to OSA subjects with a high pretest probability of OSA^7,14–18^. Prediction models reported in the literature were mostly built using clinical features including demographics (age, sex, smoking, and alcohol consumption), comorbidities, anthropometrics, OSA symptoms, physical findings, and physiologic measurements (blood pressure, overnight pulse oximetry, and pulmonary function) collected from either sleep lab- or community-based populations^7,14–20^. However, these models only use limited variables because creating a predictive model using numerous parameters including anatomical and physiological parameters may not be effective in traditional statistical models. Moreover, most prediction models for the outcome of sleep surgery tend not to have an increased sensitivity with higher specificity in providing adequate therapeutic options before treatment and it is difficult to create standardized predictive models.

Recently, machine learning, an application of artificial intelligence (AI), is the study and development of systems that can learn from and make predictions about data without the need to be programmed. Machine learning and data-mining methods enable the detection of hidden patterns in a set of data. Thus, machine learning is a potential means of addressing problems in conventional predictive modeling because of its massive parallelism, self-organization, adaptive learning capability, and robustness^21^. Hence, we considered it possible to create a good prediction model of therapeutic outcomes for sleep surgery by using machine learning methods that repeatedly analyzing the difference between the subjects who succeeded and who failed sleep surgery. The development of an objective surgical success rate prediction program using machine learning can classify OSA subjects who may be suitable for surgery and provide more effective treatment to subjects.

The present study aimed to propose an easy-to-use and accurate machine learning model to predict the surgical outcome of OSA subjects. We developed a data mining-driven prediction model using a database with features routinely collected from PSG data and the findings from anatomical and physical examinations.

## Methods

### Ethics statement

One hundred sixty-three subjects who underwent sleep surgery for diagnosed OSA at the Department of Otorhinolaryngology, Seoul National University Hospital, from March 2010 to September 2019 were recruited and analyzed retrospectively. All subjects participated in the study voluntarily and the medical records of the participants were reviewed retrospectively. Written informed consent was obtained from each participant and the study complied with the Declaration of Helsinki. The protocol of the study was approved by the Institutional Review Board of Seoul National University Hospital (IRB number: 1801-084-915).

### Subjects and study design

All subjects underwent sleep surgeries which were combined with tonsillectomy, palatal procedures, tongue base resection, and nasal surgeries to improve sleep-related symptoms and abnormal sleep parameters. Indications of sleep surgeries for OSA were (1) aged 18 years or older, (2) diagnosed as OSA (apnea–hypopnea index (AHI) ≥ 5) based on the International Classification of Sleep Disorders^22^, and (3) refused or failed PAP therapy. Among the subjects who underwent sleep surgeries, only those who had undergone physical examinations and sleep tests (PSG or home sleep apnea testing, HSAT) before and following sleep surgeries, and whose sleep parameters were available, were included. The OSA subjects who had 1) a history of previous oropharyngeal OSA surgery, 2) morbid obesity (a body mass index (BMI) > 40 kg/m^2^), 3) craniofacial abnormality, 4) other significant conditions (genetic syndrome, neuromuscular disease), or (5) AHI < 10 was excluded.

### Physical examination

All subjects underwent a preoperative upper airway examination, and the following variables were attained for analysis: septal deviation, presence of elongated uvula, tonsil size, and palate position. Tonsil size and palate position were categorized from 1 to 4 using the Friedman staging system^23^. Tonsil size grade 0 is defined as tonsillectomy status. The presence of nasal pathologies including septal deviation and inferior turbinate hypertrophy were evaluated based on intranasal endoscopic findings and confirmed it if the subjects complain of nasal obstruction and frequently breathe with the mouth open.

### Sleep study

Pre- and postoperative PSG or HSAT studies were carried out in all subjects: PSG in 110 subjects and HSAT in 63 subjects. The results of the tests were analyzed according to the scoring guidelines of the American Academy of Sleep Medicine Task Force 2007 criteria^24^. An apnea episode was defined as a complete cessation of airflow or a ≥ 90% reduction in the peak thermal sensor signal for at least 10 s. A hypopnea episode was defined as a ≥ 50% reduction in the nasal pressure signal for at least 10 s and ≥ 3% desaturation from baseline or an arousal. The mean period between preoperative PSG or HSAT and surgery was 5.6 ± 6.8 months and the mean period between sleep surgery and postoperative PSG or HSAT was about 4.1 months.

### Surgery and surgical outcomes

All subjects were treated with oropharyngeal surgery, nasal surgery, and/or hypopharyngeal surgery according to the results of their physical examination. In this study, oropharyngeal OSA surgery included uvulopalatoplasty, suspension lateral pharyngoplasty, uvuloplasty, and relocation pharyngoplasty. Nasal surgery included septoplasty, turbinoplasty, and endoscopic sinus surgery. Hypopharyngeal surgery included tongue base reduction epiglottis surgery. Objective surgical outcomes were evaluated by the result of postoperative sleep studies. Surgical success was defined as a postoperative AHI < 20 and a ≥ 50% reduction in preoperative AHI^25^. All subjects were divided into two groups: responders and non-responders. A responder was defined as a patient who had surgical success in their postoperative PSG or HSAT.

### Prediction modeling and machine learning

**Figure 1:**
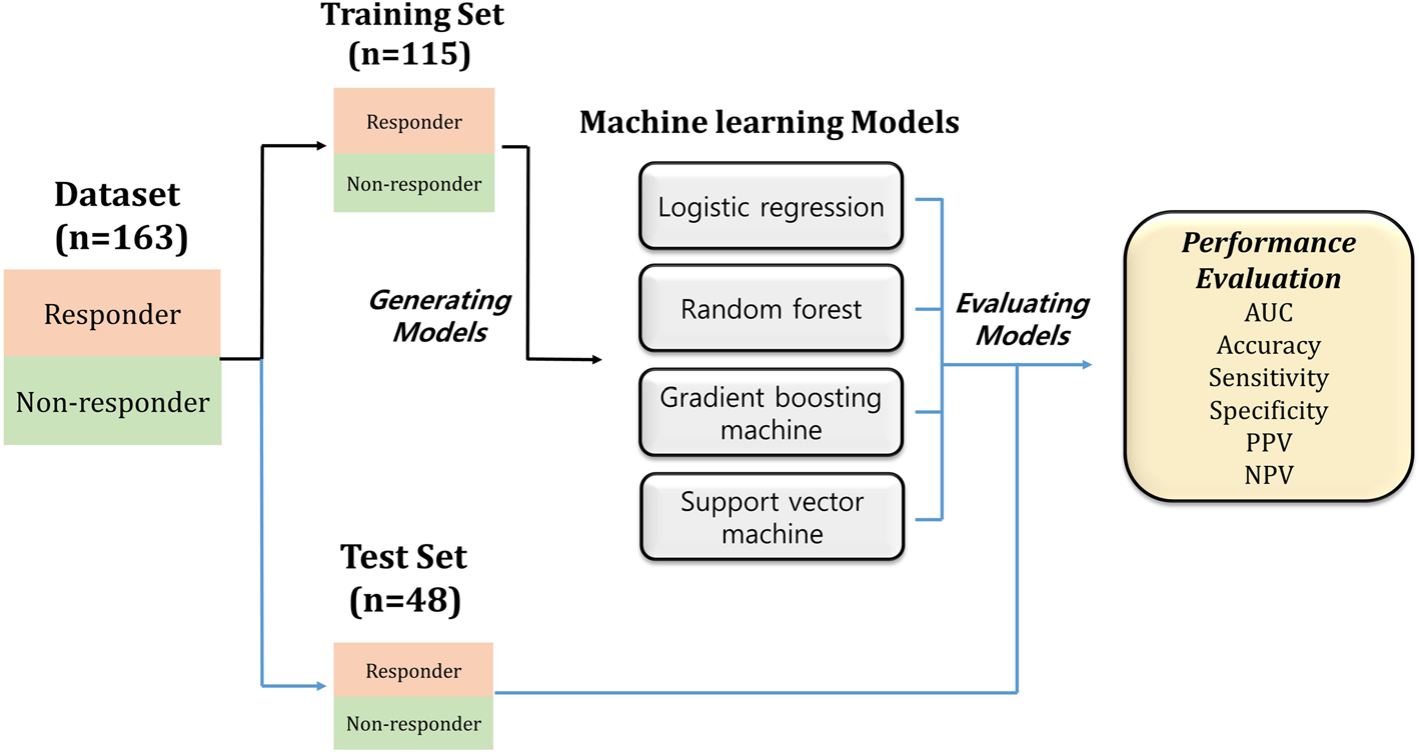
Prediction modeling and evaluation process. The total subjects (n = 163) were randomly divided into training and test sets by 7:3 ratio. The training set (n = 115) was used to derive the four prediction models: logistic regression (generalized linear model), random forest, gradient boosting machine, and support vector machine. Then, each model was applied to predict the surgical success for the subjects in test set (n = 48); surgical success was defined as a postoperative AHI < 20 and a ≥ 50% reduction in preoperative AHI. The performance of each model was evaluated by the true reference (surgical outcomes in their postoperative PSG or HSAT). *AUC* area under curve, *PPV* positive predictive value, *NPV* negative predictive value.

Fifteen variables were used for prediction models: demographic parameters (age, sex, and BMI), anatomical parameters in physical examination (presence of septal deviation, tonsil size grade, palate position grade, presence of uvula elongation, and Friedman stage), and parameters from the preoperative PSG or HSAT (preoperative AHI, ratio of non-supine versus supine AHI, sleep efficiency, percent of rapid eye movement [REM] sleep, ratio of REM versus non-rapid eye movement [NREM] sleep AHI, lowest O_2_ percent, and sleep time with oxygen saturation ≤ 90%). The subjects were randomly divided into a training set, in which the prediction models were derived, and a test set, in which the models were applied and verified: 70% of subjects were in the training set and the remaining 30% were in the testing set (Fig. 1). Considering the ratio of responders and non-responders in each set, stratified random sampling was applied. Logistic regression (generalized linear model) and three different machine learning methods (random forest, gradient boosting machine [GBM], and support vector machine [SVM]) were used to predict surgical outcomes. Random forest and GBM are ensemble models that generate a powerful model by grouping several decision trees; it can be used for both regression analysis and classification analysis^26,27^. Random forest consists of several decision trees made by randomly selecting some of the entire variables to prevent overfitting^26^. GBM is different from random forest as it generates a series of trees by emphasizing the mis-classified/predicted incidences from the previous tree^27^. SVM generates a line or hyperplane which separates the data into class while creating maximum margin between classes^28^. Hyperparameters were determined by using fivefold cross-validation and grid search on the training set to lead the best performance in the random forest, gradient boosting, and SVM models. Missing data that accounted for 1.2% of overall clinical parameters (e.g. ratio of REM versus NREM sleep AHI in a patient with undetectable REM sleep) were dealt with by multiple imputation^29^. Variable importance was determined in each model^30^.

The importance scores are used to characterize the general effect of predictors on the model. For logistic regression model, the absolute value of the t-statistic for each model parameter determines the importance scores. Based on permutation principle^31^, the differences in accuracy for permutation of each predictor variables are computed and then averaged over all trees to determine the importance score in random forest model. The importance scores in GBM are determined by using the same approach as a random forest model except that the importance scores were summed over each boosting iteration. For SVM model, receiver operating characteristic (ROC) curve analysis is conducted on each predictor, and area under the curve (AUC) is used as the measure of variable importance. A physician who specialized in otorhinolaryngology and performed OSA surgery predicted the surgical outcomes of the 163 subjects based on the 15 variables including anatomical factors and preoperative AHI, and the physician’s prediction was compared with the performance of the machine learning models. The physician predicted the surgical outcome intuitionally based on his clinical experience and had more focused on preoperative OSA severity based on AHI, and the anatomical factors of OSA subjects. If the moderate or severe OSA subjects have intranasal lesions, over grade 3 of tonsil size, and less severe retroglossal area narrowing, the surgical outcome might be predicted to be success and the physician determined the sleep surgery in the real practice.

### Statistical analysis

All statistical analyses were performed using R for Windows version 3.6.1 (R Foundation for Statistical Computing, Vienna, Austria). Library packages (caret, http://CRAN.R-project.org/package=caret) were used to obtain prediction models and variable importance^30^. Continuous variables, such as demographic parameters and PSG or HSAT data, are presented as the mean ± SD; two-sample t-tests were carried out to analyze these variables. Chi-squared tests were performed to assess categorical variables. Predicted probability of outcomes were computed for all prediction models. ROC curves were then generated to evaluate the performance of predictions of responders and non-responders confirmed by postoperative PSG or HSAT. The thresholds that yield closest points in the ROC curves to the top-left corner were selected and then used to compute the model accuracy, sensitivity, specificity, positive predictive value (PPV), and negative predictive value (NPV). As multiple classifiers are trained in a single training set and compared in a single test set, McNemar test is conducted to compare 4 machine learning classifiers. McNemar test compares the number of classifications that method A was correct while method B was incorrect to the number of classifications that method B was correct while method A was incorrect. Two sample proportion test was performed to compare the accuracy of the prediction models and the physician’s prediction. A *p*-value < 0.05 was set as the threshold for statistical significance.

## Results

### Clinical examination and sleep parameters of OSA subjects

We recruited 163 subjects who underwent sleep surgeries to resolve excessive narrowing or collapse of upper airway including nasal pathologies, palatal obstruction, tonsil hypertrophy, and tongue base narrowing. Of these, 148 subjects were men and 15 were women. The mean age was 43.5 years (range, 18–72) and the mean BMI was 26.2 kg/m^2^. Based on preoperative endoscopic findings, 90.2% of the subjects exhibited septal deviation and 98.2% showed grade 1 or larger tonsils. In addition, 94.5% of the subjects had over grade I palate position, and Friedman stage II (44.8%) or stage III (47.9%) were commonly found in the subjects. Most subjects showed anatomic narrowing at one or more structures of the upper airway and 69.3% of subjects were found to have excessive narrowing at both nasal cavity and oropharynx. The hypopharyngeal narrowing was observed not to be severe in these OSA subjects.

The severity of OSA was based on AHI and the mean value of preoperative AHI was 36.5 events/hour. Of the subjects, 19 (11.7%) had mild OSA, 51 (31.3%) had moderate OSA, and 93 (57.1%) had severe OSA. PSG findings revealed that the mean lowest O_2_ saturation was 78.1 and the mean sleep time with O_2_ saturation < 90% was 7.8% (Table 1).

**Table 1:** Clinical characteristics and management of 163 subjects.

Variables	Value, No
**Demographic parameters**	
Age, years	43.5 ± 11.8
Sex	
Male	148 (90.8%)
Female	15 (9.2%)
Body mass index, kg/m^2^	26.2 ± 3.2
**Anatomical parameters**	
Presence of septal deviation	147 (90.2%)
Tonsil size grade	
Grade 0	3 (1.8%)
Grade I	84 (51.5%)
Grade II	43 (26.4%)
Grade III	29 (17.85%)
Grade IV	4 (2.5%)
Palate position grade	
Grade I	9 (5.5%)
Grade II	56 (34.3%)
Grade III	78 (47.9%)
Grade IV	19 (11.7%)
Friedman stage	
Stage I	12 (7.4%)
Stage II	73 (44.8%)
Stage III	78 (47.9%)
Presence of uvula elongation, %	85 (52.1%)
**Parameters from the preoperative PSG or HSAT**	
Preoperative AHI, events/h	36.5 ± 19.1
Ratio of non-supine versus supine AHI	0.39 ± 0.47
Sleep efficiency, %	85.8 ± 9.4
Percent of stage REM, %	20.9 ± 7.3
Ratio of REM versus NREM AHI	1.8 ± 2.8
Lowest O2 percent, %	78.1 ± 9.8
Sleep time with oxygen saturation ≤ 90%, %	7.8 ± 13.3
**Performed surgery**	
Nasal surgery	150 (92.0%)
Oropharynx surgery	159 (97.5%)
Hypopharynx surgery	41 (33.6%)
Single-level surgery	10 (6.1%)
Multi-level surgery	153 (93.9%)

*AHI* apnea hypopnea index, *REM* rapid eye movement, *NREM* non-rapid eye movement.

Values are presented as mean ± SD or numbers (percentages).

The clinical data revealed that the correction of nasal pathologies was performed in 150 subjects (92.0%) and 159 subjects underwent palatal surgery including tonsillectomy (97.5%). The rate of anatomic narrowing in tongue base and hypopharynx was relatively lower and only 33.6% of subjects underwent hypopharynx surgery such as tongue base resection and partial epiglottectomy (Table 1). Because multi-level narrowing of upper airway was commonly found in 163 subjects, 153 subjects underwent multi-level surgery (93.9%), and only 10 subjects underwent single-level surgery (6.1%). Among the 153 subjects who underwent multi-level surgery, 147 had multi-level surgery including both nasal and palatal surgeries with or without hypopharynx surgery. The success rate of sleep surgery was determined using pre- and post-operative sleep parameters and the overall severity of OSA improved significantly after sleep surgeries (36.5 versus 21.5, *p* < 0.001) in 163 subjects. PSG data revealed that 80 subjects were classified into the responder group and 83 into the non-responder group.

### Comparison of sleep parameters and clinical factors between responders and non-responders

Next, we compared the demographic data, clinical findings from physical examination, and the sleep parameters between responders and non-responders to determine the factors that can predict the surgical outcome of OSA subjects (Table 2). There was a significant difference BMI, the presence of nasal pathologies, tongue size grade, preoperative AHI, lowest O_2_ saturation, and sleep time with oxygen saturation ≤ 90% between the two groups.

**Table 2:** Comparison of clinical parameters between responder and non-responder groups.

Variables	Responder (n = 80)	Non-responder (n = 83)	*P*-value
**Demographic parameters**			
Age, years	42.1 ± 11.7	44.8 ± 11.8	0.147
Sex, male: female	72:8	76:7	0.940
Body mass index, kg/m2	25.7 ± 3.1	26.8 ± 3.3	**0.033**
**Anatomical parameters**			
Presence of Septal deviation, %	67/80 (83.8%)	80/83 (96.4%)	**0.014**
Tonsil size grade	1.9 ± 0.9	1.5 ± 0.8	**0.007**
Palate position grade	2.6 ± 0.8	2.7 ± 0.7	0.137
Friedman stage	2.3 ± 0.6	2.5 ± 0.6	0.110
Presence of uvula elongation, %	41/80 (51.3%)	44/83(53.0%)	0.746
**Parameters from the preoperative PSG or HSAT**			
Preoperative AHI, events/h	33.0 ± 17.3	39.9 ± 20.2	**0.020**
Ratio of non-supine versus supine AHI	0.41 ± 0.58	0.37 ± 0.34	0.671
Sleep efficiency, %	86.6 ± 8.7	85.1 ± 10.1	0.318
Percent of stage REM, %	20.9 ± 7.8	20.9 ± 6.9	0.998
Ratio of REM versus NREM AHI	2.0 ± 3.3	1.7 ± 2.0	0.530
Lowest O2 percent, %	80.9 ± 9.0	75.5 ± 9.8	**< 0.001**
Sleep time with oxygen saturation ≤ 90%, %	4.2 ± 7.4	11.2 ± 16.4	**< 0.001**

Bold values indicates statistical significance (*P* < 0.05).

AHI, apnea hypopnea index; REM, rapid eye movement; NREM, non-rapid eye movement.

Values are presented as mean ± SD or numbers (percentages).

We found that the mean BMI of the responders was lower than that of non-responders. On the comparison of anatomical parameters, the degree of nasal septal curvature was more severe in non-responders and the mean tonsil grade was higher in responders. Comparing the parameters from the preoperative PSG, the responders had a higher preoperative AHI than non-responders and the lowest O_2_ saturation was relatively higher and sleep time with oxygen saturation ≤ 90% was lower in the responder group. Through these clinical data, we were able to determine the clinical factors that showed a significant difference between responders and non-responders, and we presume that preoperative analysis of these data through machine learning might be effective to predict therapeutic outcome of sleep surgery.

### Prediction of surgical outcome using machine learning

**Figure 2:**
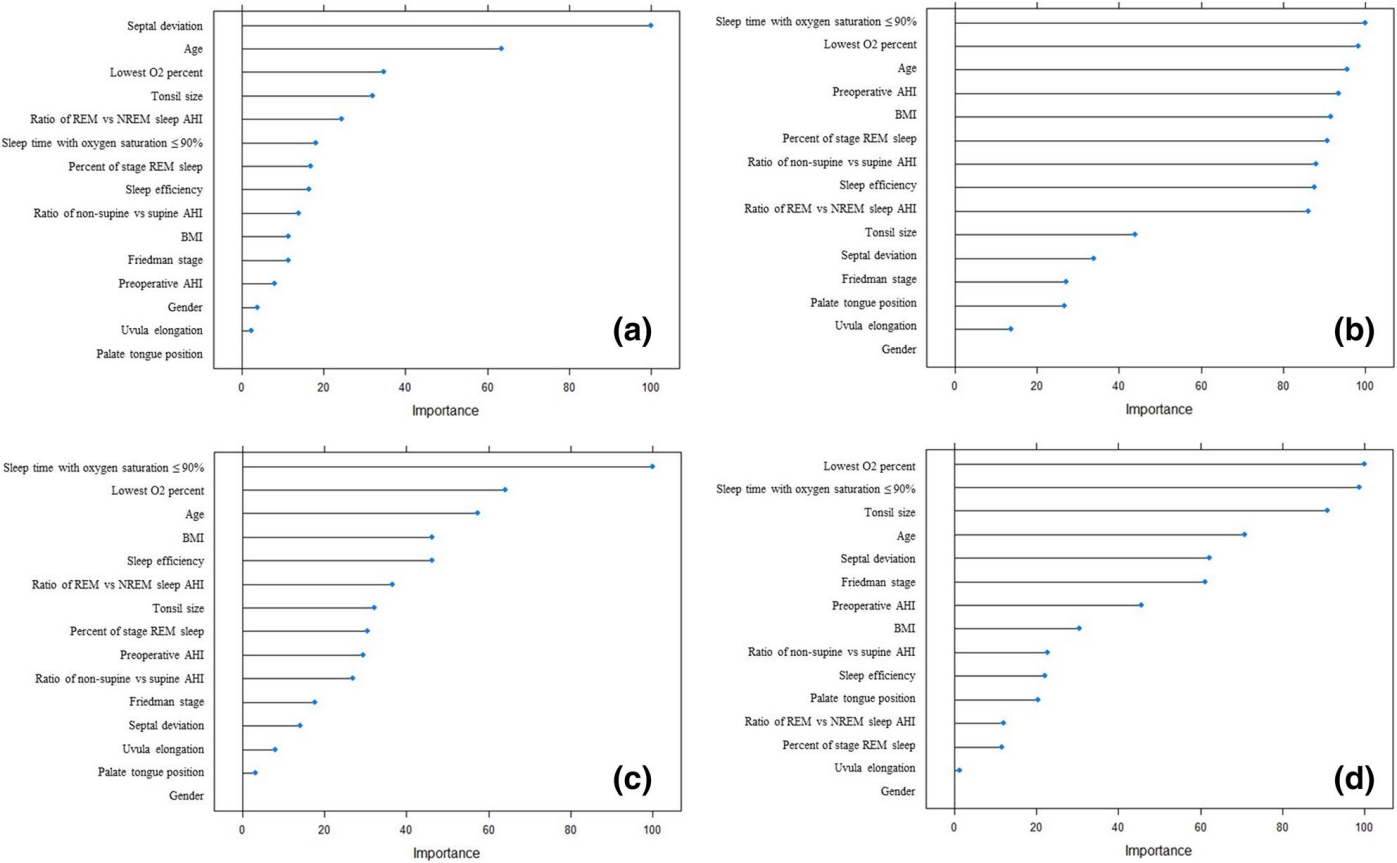
Importance of the variables in each model. (**a**) Logistic regression, (**b**) random forest, (**c**) gradient boosting, and (**d**) support vector machine. *AHI* apnea–hypopnea index, *BMI* Body mass index, *REM* rapid eye movement, *NREM* non-rapid eye movement.

**Figure 3:**
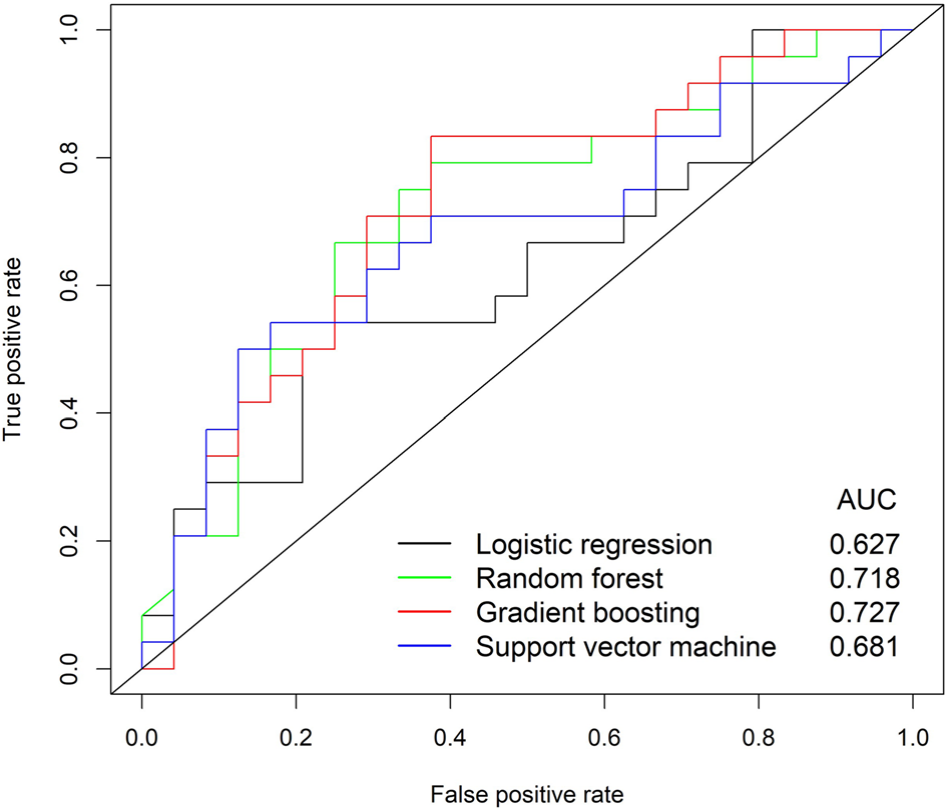
Receiver operating characteristic (ROC) curves for machine learning models. Area under the curve (AUC) are shown for each model. The color of lines in the ROC curve represents each algorithm; logistic regression (black); random forest (green); gradient boosting (red); support vector machine (blue). GBM, gradient boosting machine; SVM, support vector machine.

Among the 163 subjects, 115 (70.6%) subjects were randomly assigned to the training set and the remaining 48 (29.4%) were randomly assigned to the testing set. To predict surgical outcomes, logistic regression and three machine learning models were derived by using the data from the training set: Logistic regression (Fig. 2a), random forest (Fig. 2b), gradient boosting (Fig. 2c), and SVM (Fig. 2d) models. Variable importance in each model shows the relative importance of the parameters in predicting the surgical outcome. The variable importance is measured in a scale from 0 to 100 with the most important variable having a value of 100 and the least important variable having a value of 0. In the logistic regression, septal deviation, age, lowest O_2_ level, and tonsil size were major contributors to surgical outcomes (Fig. 2a). Sleep time with oxygen saturation less than 90%, lowest O_2_ level, and age were major contributors in the other models (Fig. 2b-2d). BMI was also a major contributor in the random forest and gradient boosting models (Fig. 2b,c). In addition, tonsil size and septal deviation were ranked highly as important variables in the random forest model (Fig. 2b). The majority of important contributors in the four machine learning models correspond to the significant parameters in the comparison analysis between responder and non-responder groups: BMI, presence of septal deviation, tonsil size, preoperative AHI, sleep time with oxygen saturation ≤ 90%, and lowest O_2_ percent. However, some parameters such as age and sleep efficiency, which are not significant in the comparison analysis between responder and non-responder groups, also seem to play an important role in the machine learning models. The analytic data generated a receiver operating characteristic (ROC) curve for the prediction of surgical outcomes in OSA subjects; the area under the curve (AUC) was highest in the gradient boosting model (Fig. 3). In addition, Table 3 represents the accuracy, sensitivity, specificity, and positive predictive value, negative predictive value for each model. The gradient boosting model showed the highest accuracy, while the logistic regression model showed the lowest accuracy. In contrast to the traditional model (logistic regression model) that does not analyze the complex interaction between variables, machine learning models analyze the interaction and the association between variables, which may improve their predictive performance. Table 3 represents accuracy, sensitivity, specificity, positive predictive value for each model. Gradient boosting model showed higher accuracy than logistic regression model (*p* = 0.033; Fig. 3). Although significance was not met, random forest and SVM models had higher accuracy than logistic regression model (*p* = 0.083 and *p* = 0.109, respectively). The physicians predicted surgical outcomes in a full dataset of 163 participants based on 15 clinical parameters used in the prediction models (Table 3), and the performance of the physician’s prediction and the prediction models was also compared. Although there was no significant difference in accuracy between the physician’s prediction and the logistic regression model (*p* = 0.467), the machine learning models had a higher performance in accuracy than the physician’s prediction (*p* = 0.053, 0.017, and 0.053 in the random forest, gradient boosting, and SVM models, respectively).

**Table 3:** Performance metrics of machine learning models and physician’s prediction.

	Accuracy	Sensitivity	Specificity	PPV	NPV
Logistic regression	0.542 (0.400–0.683)	0.417 (0.277–0.556)	0.667 (0.533–0.800)	0.556 (0.415–0.696)	0.533 (0.392–0.674)
Random forest	0.667 (0.533–0.800)	0.542 (0.400–0.683)	0.792 (0.677–0.907)	0.722 (0.595–0.849)	0.633 (0.497–0.770)
Gradient boosting	0.708* (0.580–0.837)	0.708 (0.580–0.837)	0.708 (0.580–0.837)	0.708 (0.580–0.837)	0.708 (0.580–0.837)
Support vector machine	0.667 (0.533–0.800)	0.708 (0.580–0.837)	0.625 (0.488–0.762)	0.654 (0.519–0.788)	0.682 (0.550–0.814)
Physician’s prediction	0.522 (0.380–0.663)	0.238 (0.117–0.358)	0.795 (0.681–0.909)	0.528 (0.387–0.669)	0.520 (0.378–0.661)

*PPV* positive predictive value, *NPV* negative prediction value.

*p < 0.05 compared with logistic regression or physician’s prediction.

The values were presented as mean (95% confidence interval).

## Discussion

Here, we found that the therapeutic outcomes of sleep surgeries could be predicted prior to surgery in OSA subjects by using machine learning models based on the demographic features, sleep parameters, and the characteristics of subjects’ upper airway structures. Our data also suggest that the predicted surgical outcome from preoperative machine learning might provide more adequate therapeutic options to OSA subjects in concert with avoidance of unnecessary sleep surgery.

Surgical modification of the upper airway is suitable for select subjects and is often recommended for symptomatic subjects unable to tolerate PAP therapy. There are many discrepancies in the literature related to the therapeutic outcome of surgical intervention in OSA, and it is clinically important to recommend sleep surgery to OSA subjects who are judged to have higher success rate^10–12^. Until now, favorable candidates for sleep surgery may be decided thorough preoperative evaluation including endoscopic examination of upper airway, drug-induced sleep endoscopy, cephalometry, and PSG. The predicted success rate of sleep surgery was somewhat inaccurate and was determine by sleep surgeon’s subjective clinical experience. It is widely accepted that more method for objective surgical outcome prediction would be needed and the comparison of clinical factors in OSA subjects should be focused on detecting the distinctive points between responders and non-responders to predict success rate prior to sleep surgery^32–35^. The mean age of the subjects in this study was 43.5 years that may be low considering the prevalence of OSA peaks at approximately 55 years of age^36^. However, as many young OSA subjects are reluctant to use PAP every night and seek for surgical treatment due to low compliance on PAP, the age of subjects who underwent OSA surgery in other studies^7,14,15^. In addition, the ratio of male and female subjects (9:1) in the present study was higher than that reported in the previous study^36^ (ranges between 2:1 and 4:1). Therefore, the population who underwent OSA surgery in this study might be young and male-dominated population compared with general population of OSA. For surgical method, as upper airway obstruction was found at multiple sites, most subjects underwent multi-level surgery. The surgical method was not used as the parameters deriving machine learning models, as preoperative anatomical structures determine the surgical method. However, the difference in the detail surgical technique and skillfulness between the surgeons were not considered, because these factors are too complicated and difficult to be quantified.

We also compared clinical factors including sleep parameters and anatomic structures of the upper airway in OSA subjects between responders and non-responders. Our data showed that the mean value of BMI was relatively lower in responders and the success rate of sleep surgery was significantly lower in obese OSA subjects.

The current data also revealed that the presence of nasal pathologies might be closely related to the success or failure of surgery in OSA subjects and the surgical correction of nasal pathologies would be helpful better outcome of sleep surgery. In addition, tonsil grade in the responders was higher than that of non-responders and the OSA subjects with relatively larger tonsils exhibited a higher success rate after sleep surgery.

Sleep parameters still represent essential factors in determining the therapeutic outcome of OSA subjects. Evidently, the difference in sleep parameters before therapeutic trials in OSA subjects seems to predict the success rate of OSA treatment including sleep surgery^7,14^. In the present study, preoperative AHI was significantly lower and the lowest O_2_ saturation was relatively higher in responders. In addition, sleep time with oxygen saturation ≤ 90% was significantly longer in non-responders than in responders. In the present study, postoperative complications were not assessed due to its retrospective nature. Moreover, it was difficult to quantify the subject's subjective symptoms, and the rate of serious complications lasting 4 to 6 months after sleep surgery was very minimal, so we presumed that the occurrence of complications or side effects of surgery did not significantly affect the results of polysomnography performed 4 months later.

Next, we conducted a study to establish an appropriate machine learning model using clinical factors and sleep parameters of OSA subjects. The data processing in machine learning is different from traditional statistical analysis. In traditional statistical approaches, researchers commonly choose a pre-designed model that is most appropriate for the data to predict surgical outcome of sleep surgery. Thus, the major limitation of the traditional statistical model is that only theoretically relevant parameters based on previous studies and experience, or significant parameters in the univariate analysis are used. In addition, the studies related to sleep surgery that have already been published were more focused on anatomic factors to evaluate therapeutic outcome and many variables in PSG or HSAT had not been used to predict therapeutic outcomes of sleep surgery^2,14,15^.

In contrast, machine learning is not created on a pre-structured model; instead, the numerous variables in data produce the model by detecting the underlying patterns. This approach prevents the pre-assumptions regarding types of models and interaction between variables. Considering these analytic advantages, machine learning may find the hidden knowledge that remains undetected by conventional statistical analysis. In the present study, logistic regression and three different machine learning methods were used to predict the surgical outcomes of sleep surgery in OSA subjects, and the predicted surgical outcome of sleep surgery was calculated in each machine learning model based on sleep parameters and the characteristics of subjects’ upper airway structures. Although the importance of variables was different between the models, sleep time with oxygen saturation less than 90% and lowest O_2_ level were highly ranked variables across all the models. In particular, sleep time with oxygen saturation less than 90% and the lowest O_2_ were the highest ranked variables in the random forest, gradient boosting machine, and SVM models. Because sleep surgery corrects anatomical structures in the upper airway of OSA subjects, surgeons often underestimated other physiological factors in sleep studies except for preoperative AHI. However, the consistent importance of sleep time with oxygen saturation less than 90% and lowest O_2_ saturation suggest the importance of preoperative sleep parameters for sleep surgery. Among the four machine learning models, gradient boosting shows the best performance in AUC, accuracy, sensitivity, specificity, and positive predictive value to predict surgical outcomes; the lowest performance was by the logistic regression model. The performance of the gradient boosting model had higher accuracy than the physician’s prediction and logistic regression model, which indicates the possible clinical application of machine learning in the prediction of the clinical outcomes of sleep surgery. The physician expected many surgical failures due to high AHI, therefore, the physician’s prediction showed low sensitivity. Feature selection strategies using information gain, gain ratio, and recursive feature elimination methods were performed. However, it did not improve the performance of the models and the performance of the gradient boosting model was even lower with selected features (data not shown). Therefore, we used all the variables to derive machine learning models.

We estimate that the complex interactions between the anatomical and physiologic variables are the obstacles in predicting surgical outcome properly because both anatomical and physiologic factors contribute to the pathophysiology of OSA^37^. Using a machine learning model, more variables which were representative of both anatomical and physiologic parameters were used properly to derive prediction models than those in a conventional statistical model such as a logistic regression model that used in previous studies^7,14,15^.

Moreover, the machine learning approach elicits a better prediction model by detecting previously unknown associations between anatomical variables and sleep parameters. In particular, the subjects were divided into a training set and test set in the present study, and the machine learning models derived from the training set were applied to the different cohort (test set), which predicted more acceptable real surgical outcomes. Recently, clustering analysis, unsupervised machine learning, has been performed to investigate phenotypes of OSA^38,39^. Clustering analysis showed different phenotypes of OSA through the various clinical clusters^38,39^. The present study aimed to predict surgical outcomes immediately for candidates for sleep surgery in OSA subjects and used supervised machine learning techniques, which may be helpful for especially inexperienced surgeons when determining OSA surgery. However, a clustering analysis may be helpful to find a distinct phenotype of OSA with high surgical success rate in the future.

There are some limitations in this study. First, subjects were obtained from two different sleep studies (PSG and HSAT). Therefore, some parameters (e.g. central apnea) which are only available in PSG were not utilized to derive machine learning models. Further investigation with more OSA subjects who underwent preoperative and postoperative PSG is necessary. Second, revision surgery was not evaluated for consistency of the preoperative status. Hence, these models were only applicable in the subjects without previous history of oropharyngeal OSA surgery. Third, the clinical data analyzed in this study do not represent general population for OSA subjects in terms of age and sex ratio. However, the difference in age and sex ration between the general population of OSA and that of patient who underwent OSA surgery is also reported in the previous studies^7,14,15^. Lastly, the time point determining the surgical outcome through postoperative PSG or HSAT might affect the actual surgical outcome. For the subject's personal circumstances, we performed PSG or HSAT at an average of 4 months after sleep surgery, but we have not been able to analyze the difference in the subject's sleep factors at each time point of PSG or HSAT or apply them to machine learning methods. It was more adequate to proceed with PSG or HSAT at each time point in a group of OSA patients with the same other conditions, but it was impossible to recruit those OSA subjects in a retrospective study using clinical data. The time point determining the surgical outcome should be controlled in the further study with prospective design.

In summary, machine learning models such as the gradient boosting model can supply the accurate prediction about the surgical outcome of sleep surgery based on demographics, anatomical characteristics, and sleep parameters prior to sleep surgery. The predicted surgical outcome from machine learning-derived analysis might provide a key clinical decision for adequate therapeutic options of OSA.

## References

[1] Koutsourelakis I, Safiruddin F, Ravesloot M, Zakynthinos S, de Vries N (2012). Surgery for obstructive sleep apnea: Sleep endoscopy determinants of outcome. Laryngoscope.

[2] Braga A (2013). Predictors of uvulopalatopharyngoplasty success in the treatment of obstructive sleep apnea syndrome. Sleep Med..

[3] Lin H-S (2007). Treatment compliance in patients lost to follow-up after polysomnography. Otolaryngol. Head Neck Surg..

[4] Woodson BT (2008). Structural effectiveness of pharyngeal sleep apnea surgery. Sleep Med. Rev..

[5] Franklin KA (2009). Effects and side-effects of surgery for snoring and obstructive sleep apnea—A systematic review. Sleep.

[6] Robinson S (2009). Upper airway reconstructive surgery long-term quality-of-life outcomes compared with CPAP for adult obstructive sleep apnea. Otolaryngol. Head Neck Surg..

[7] Li Y (2017). Physiology-based modeling may predict surgical treatment outcome for obstructive sleep Apnea. J. Clin. Sleep Med..

[8] Haniffa, M., Lasserson, T. J. & Smith, I. Interventions to improve compliance with continuous positive airway pressure for obstructive sleep apnoea. *The* Cochrane Database Syst Rev, Cd003531 (2004). 10.1002/14651858.CD003531.pub2 15495057

[9] Sutherland K (2015). Oral appliance treatment response and polysomnographic phenotypes of obstructive sleep apnea. J. Clin. Sleep Med..

[10] Friedman M, Lin HC, Gurpinar B, Joseph NJ (2007). Minimally invasive single-stage multilevel treatment for obstructive sleep apnea/hypopnea syndrome. Laryngoscope.

[11] Richard W, Kox D, den Herder C, van Tinteren H, de Vries N (2007). One stage multilevel surgery (uvulopalatopharyngoplasty, hyoid suspension, radiofrequent ablation of the tongue base with/without genioglossus advancement), in obstructive sleep apnea syndrome. Eur. Arch. Otorhinolaryngol..

[12] Lin HC, Friedman M, Chang HW, Gurpinar B (2008). The efficacy of multilevel surgery of the upper airway in adults with obstructive sleep apnea/hypopnea syndrome. Laryngoscope.

[13] Epstein LJ (2009). Clinical guideline for the evaluation, management and long-term care of obstructive sleep apnea in adults. J. Clin. Sleep Med..

[14] Zhang J (2014). The combination of anatomy and physiology in predicting the outcomes of velopharyngeal surgery. Laryngoscope.

[15] Choi JH (2017). Predictive models of objective oropharyngeal OSA surgery outcomes: Success rate and AHI reduction ratio. PLoS ONE.

[16] Petri N (2019). Mandibular advancement device therapy for obstructive sleep apnea: A prospective study on predictors of treatment success. Sleep Med..

[17] Ebben MR, Narizhnaya M, Krieger AC (2017). A new predictive model for continuous positive airway pressure in the treatment of obstructive sleep apnea. Sleep Breath.

[18] Riachy M (2017). Factors predicting CPAP adherence in obstructive sleep apnea syndrome. Sleep Breath.

[19] Endeshaw YW, White WB, Kutner M, Ouslander JG, Bliwise DL (2009). Sleep-disordered breathing and 24-hour blood pressure pattern among older adults. J. Gerontol. A Biol. Sci. Med. Sci..

[20] Gottlieb DJ, Yao Q, Redline S, Ali T, Mahowald MW (2000). Does snoring predict sleepiness independently of Apnea and hypopnea frequency?. Am. J. Respir. Crit. Care Med..

[21] Jiang F (2017). Artificial intelligence in healthcare: Past, present and future. Stroke Vasc. Neurol..

[22] Medicine, A. A. O. S. The AASM manual for the scoring of sleep and associated events: rules, terminology and technical specifications. *Westchester, IL: American Academy of Sleep Medicine*, 23 (2007).

[23] Friedman M, Ibrahim H, Bass L (2002). Clinical staging for sleep-disordered breathing. Otolaryngol. Head Neck Surg..

[24] Berry RB (2012). Rules for scoring respiratory events in sleep: update of the 2007 AASM manual for the scoring of sleep and associated events. J. Clin. Sleep Med..

[25] Sher AE, Schechtman KB, Piccirillo JF (1996). The efficacy of surgical modifications of the upper airway in adults with obstructive sleep apnea syndrome. Sleep.

[26] Breiman L (2001). Random forests. Mach. Learn..

[27] Kwon H, Park J, Lee Y (2019). Stacking ensemble technique for classifying breast cancer. Healthc. Inform. Res..

[28] Noble WS (2006). What is a support vector machine?. Nat. Biotechnol..

[29] Royston P (2004). Multiple imputation of missing values. Stata J..

[30] Kuhn M (2008). Building predictive models in R using the caret package. J. Stat. Softw..

[31] Strasser, H. & Weber, C. On the asymptotic theory of permutation statistics. (1999).

[32] Bradford A, McGuire M, O’Halloran KD (2005). Does episodic hypoxia affect upper airway dilator muscle function? Implications for the pathophysiology of obstructive sleep apnoea. Respir. Physiol. Neurobiol..

[33] Won CH, Li KK, Guilleminault C (2008). Surgical treatment of obstructive sleep apnea: upper airway and maxillomandibular surgery. Proc. Am. Thorac Soc..

[34] Com G (2015). Characteristics and surgical and clinical outcomes of severely obese children with obstructive sleep apnea. J. Clin. Sleep Med..

[35] Grewal G, Joshi GP (2019). Obesity and obstructive sleep apnea in the ambulatory patient. Anesthesiol. Clin..

[36] Lurie, A. In *Obstructive Sleep Apnea in Adults* Vol. 46 1–42 (Karger Publishers, 2011).

[37] Woodson BT (1999). Predicting which patients will benefit from surgery for obstructive sleep apnea: The ENT exam. Ear Nose Throat J..

[38] Keenan BT (2018). Recognizable clinical subtypes of obstructive sleep apnea across international sleep centers: A cluster analysis. Sleep.

[39] Kim J-W (2020). Polysomnographic phenotyping of obstructive sleep apnea and its implications in mortality in Korea. Sci. Rep..

